# Complete Absence of Menstruation

**Published:** 1841-01

**Authors:** 


*Complete absence of Menstruation.* By M. Kruger-Hansen.—A woman of about fifty-eight years of age, of a robust constitution, and who had always enjoyed good health, much younger in appearance than she really was, and in whom all the feminine characters were well developed, never in the whole course of her life had any discharge at all similar to the menses. She never had *flour albus*, or any abnormal sanguineous discharges or sweats, to compensate for the want of the usual monthly secretion. It is added that the sexual appetite was present, but that she never had borne children. M. Kruger-Hansen, from this single imperfect case, draws the conclusion that the menstrual discharge is not absolutely necessary to woman. It is obvious, however, that, before such a conclusion could be drawn, it would be necessary to ascertain whether all the organs were present, and well developed. The omission of this renders the case incomplete. *Graefe's and Walter's Journal. Ib.*

